# Serial amyloid PET demonstrates marked reduction in amyloid burden following donanemab treatment: a real-world cohort study

**DOI:** 10.1007/s11604-026-02054-x

**Published:** 2026-07-27

**Authors:** Yoichi Otomi, Takayoshi Shinya, Hideki Otsuka, Hiroto Kasai, Tomoki Matsushita, Yukiko Takaoka, Yuka Hiroshima, Tomoyasu Matsubara, Koji Fujita, Yukiko Tomioka, Masahito Nakataki, Shusuke Numata, Yuishin Izumi, Masafumi Harada

**Affiliations:** 1 Department of Radiology, Tokushima University Hospital, Tokushima, Japan; 2 Department of Neurology, Tokushima University Graduate School of Biomedical Sciences, Tokushima, Japan; 3 Department of Psychiatry, Tokushima University Graduate School of Biomedical Sciences, Tokushima, Japan

**Keywords:** Amyloid PET, Donanemab, Alzheimer’s disease, Centiloid, Treatment response

## Abstract

**Purpose:**

To evaluate changes in amyloid burden on serial amyloid PET before and after donanemab treatment in a real-world clinical cohort.

**Methods:**

This retrospective study included 20 consecutive patients who underwent serial amyloid PET before and after donanemab treatment. Quantitative analysis was performed using Centiloid values and tracer-specific standardized uptake value ratios (SUVr). Visual assessment was also performed to determine amyloid-positive or amyloid-negative status before and after treatment.

**Results:**

Centiloid values significantly decreased from 52.3 ± 23.4 at baseline to − 5.6 ± 16.5 at follow-up, with a mean change of − 58.0 (*p* < 0.001). All patients showed a reduction in Centiloid values. In the flutemetamol group (n = 12), pons-referenced SUVr significantly decreased, while in the florbetapir group (n = 8), cerebellar-referenced SUVr also showed significant reductions (both *p* < 0.001). On visual assessment, 17 of 20 patients (85%) converted from amyloid-positive to amyloid-negative status after treatment.

**Conclusion:**

Serial amyloid PET demonstrated a substantial reduction in amyloid burden following donanemab treatment in this real-world cohort. Quantitative amyloid PET, particularly using Centiloid scaling, may be a useful tool for monitoring treatment response in clinical practice.

**Supplementary Information:**

The online version contains supplementary material available at https://doi.org/10.1007/s11604-026-02054-x.

## Introduction

Alzheimer’s disease is characterized by the accumulation of β-amyloid in the brain, and amyloid PET imaging has become an essential tool for its in vivo detection and quantification [1–3]. It is now considered a key biomarker in the biological definition of Alzheimer’s disease [4]. Recent revisions have further refined the biological diagnosis and staging framework for Alzheimer's disease [5]. The clinical application of amyloid PET has been further supported by established appropriate use criteria [6]. Recent advances in disease-modifying therapies targeting amyloid have highlighted the importance of imaging biomarkers for evaluating treatment response.

Donanemab, an anti-amyloid monoclonal antibody, has demonstrated significant reductions in amyloid burden in clinical trials, including the TRAILBLAZER-ALZ study and the TRAILBLAZER-ALZ 2 study [7, 8]. These studies have shown that quantitative amyloid PET, particularly using the Centiloid scale, can sensitively detect treatment-related changes in amyloid deposition. The Centiloid scale provides a standardized method for quantifying amyloid burden across different tracers and imaging platforms [9]. Although no universally established cutoff exists for amyloid positivity on the Centiloid scale, values around 20 have been commonly used as a practical threshold in previous studies [10].

Despite these advances, data on serial amyloid PET findings in routine clinical practice remain limited. In particular, real-world evidence demonstrating consistent changes in amyloid burden using quantitative PET metrics is still scarce. Furthermore, differences in tracers and reference regions complicate the interpretation of standardized uptake value ratios (SUVr), underscoring the importance of standardized metrics such as Centiloid.

In this study, we aimed to evaluate changes in amyloid burden using serial amyloid PET before and after donanemab treatment in a real-world clinical cohort. We assessed both Centiloid values and tracer-specific SUVr to determine the consistency and magnitude of treatment-related changes.

## Methods

### Patients

This retrospective study included 20 consecutive patients with cognitive impairment who underwent serial amyloid PET imaging before and after treatment with donanemab. All patients were clinically diagnosed with Alzheimer’s disease or suspected Alzheimer’s disease based on standard clinical criteria.

The interval between baseline and follow-up PET scans was 11.7 ± 0.3 months. This retrospective study was approved by the Ethics Committee of Tokushima University Hospital (Approval No. 4588), and the requirement for written informed consent was waived due to the retrospective nature of the study. An opt-out approach was implemented through the institutional website in accordance with the institutional ethics policy.

### PET acquisition

Amyloid PET imaging was performed using either ^18^F-flutemetamol (Vizamyl®, GE Healthcare, Chicago, IL, USA) or ^18^F-florbetapir (Amyvid®, Eli Lilly and Company, Indianapolis, IN, USA) according to clinical availability. PET/CT scans were acquired using a Discovery 710/128 system (GE Healthcare, Chicago, IL, USA). For ^18^F-florbetapir, a dose of 370 MBq was administered, and image acquisition was performed 50 min after injection for 20 min. For ^18^F-flutemetamol, a dose of 185 MBq was administered, and image acquisition was performed 90 min after injection for 20 min. Images were reconstructed using an ordered subset expectation maximization algorithm with Q.Clear (GE Healthcare, Chicago, IL, USA), incorporating corrections for attenuation using CT-based attenuation correction (CTAC), scatter (model-based), and random coincidences (single-event method). Reconstruction was performed with a matrix size of 128 × 128, a pixel size of 2.34 × 2.34 mm, and a slice thickness of 3.27 mm. The Q.Clear parameter was set to β = 500.

### Image analysis

Quantitative analysis consisted of two separate components: tracer-specific SUVr analysis and Centiloid analysis. Tracer-specific SUVr values were calculated using CortexID Suite (GE Healthcare, Chicago, IL, USA). For ^18^F-flutemetamol, pons-referenced SUVr values were obtained, whereas for ^18^F-florbetapir, cerebellar-referenced SUVr values were obtained.

Centiloid values were calculated independently using tracer-specific validated software pipelines. For ^18^F-flutemetamol, Centiloid values were calculated using VIZCalc (Nihon Medi-Physics, Tokyo, Japan), which uses a whole cerebellum reference region for Centiloid conversion [11, 12]. For ^18^F-florbetapir, Centiloid values were calculated using AMYclz Neuro (PDR Pharma, Tokyo, Japan), which provides standardized Centiloid values based on an established anatomical standardization and quantification pipeline [13]. Centiloid values were used as the primary quantitative measure to enable comparison across tracers. These calculations were performed independently from the tracer-specific SUVr analyses.

### Visual assessment

Visual assessment of amyloid PET images was performed independently by two experienced nuclear medicine physicians in accordance with established tracer-specific visual interpretation criteria for ^18^F-flutemetamol and ^18^F-florbetapir [6, 14]. All readers had completed the official amyloid PET interpretation training program offered by the Japanese Society of Nuclear Medicine. Baseline and follow-up PET scans were reviewed side-by-side during image interpretation to assess treatment-related changes. Although quantitative measures, including Centiloid values, were available as part of routine clinical practice, the final classification was based primarily on visual assessment rather than predefined quantitative thresholds. Discrepancies were resolved by consensus.

### Statistical analysis

Continuous variables are presented as mean ± standard deviation. Changes in Centiloid values and SUVr between baseline and follow-up were assessed using paired t-tests. Because SUVr values were derived using different reference regions depending on tracer type, pooled SUVr analysis across all cases was not performed. Instead, SUVr changes were analyzed separately for each tracer group. A *p* value < 0.05 was considered statistically significant. All statistical analyses were performed using SPSS (version 29; IBM Corp., Armonk, NY, USA).

A large language model (ChatGPT, OpenAI, San Francisco, CA, USA) was used solely to assist in language refinement of the manuscript under human supervision. The model was not used for data analysis or interpretation.

## Results

**Fig. 1:**
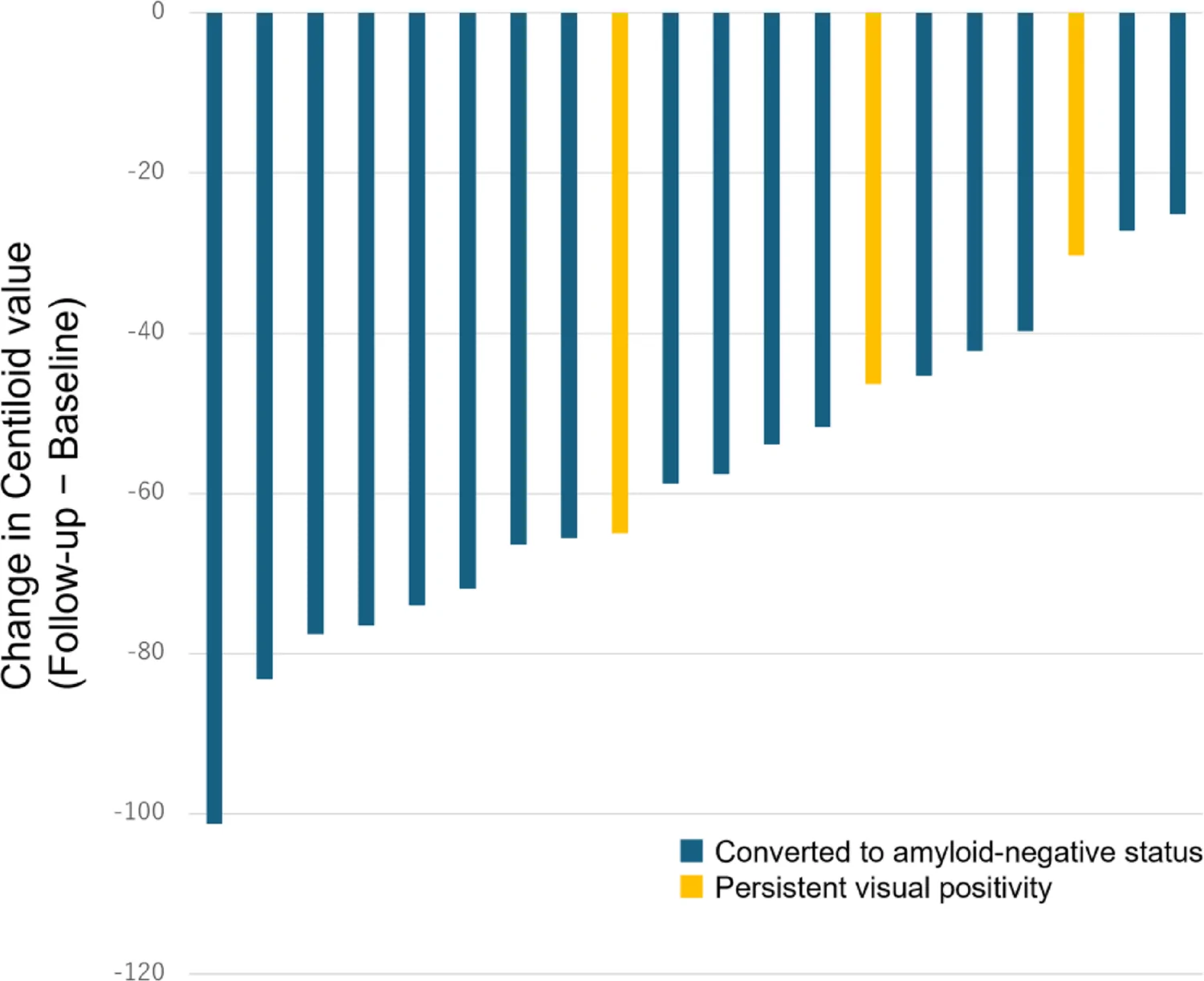
Waterfall plot showing individual changes in Centiloid values following donanemab treatment. Each bar represents the change in Centiloid value (follow-up minus baseline) for an individual patient. Blue bars indicate patients who converted from visually amyloid-positive to amyloid-negative status, whereas orange bars indicate patients with persistent visual amyloid positivity at follow-up

**Fig. 2:**
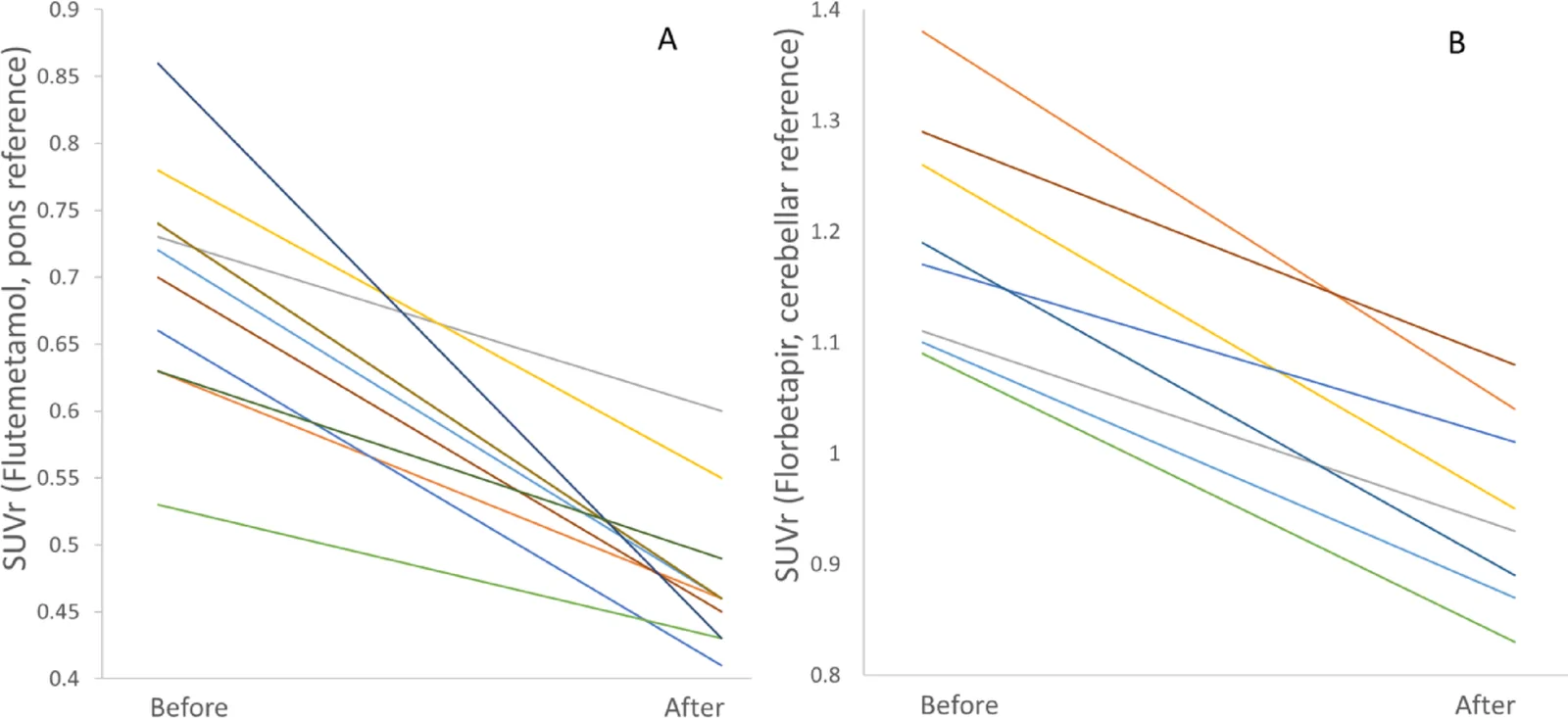
Changes in standardized uptake value ratios (SUVr) before and after donanemab treatment. **A** Flutemetamol PET using the pons as a reference region (n = 12). **B** Florbetapir PET using the cerebellum as a reference region (n = 8). SUVr values were analyzed separately due to differences in reference regions. All patients showed a reduction in SUVr in both tracer groups

**Fig. 3:**
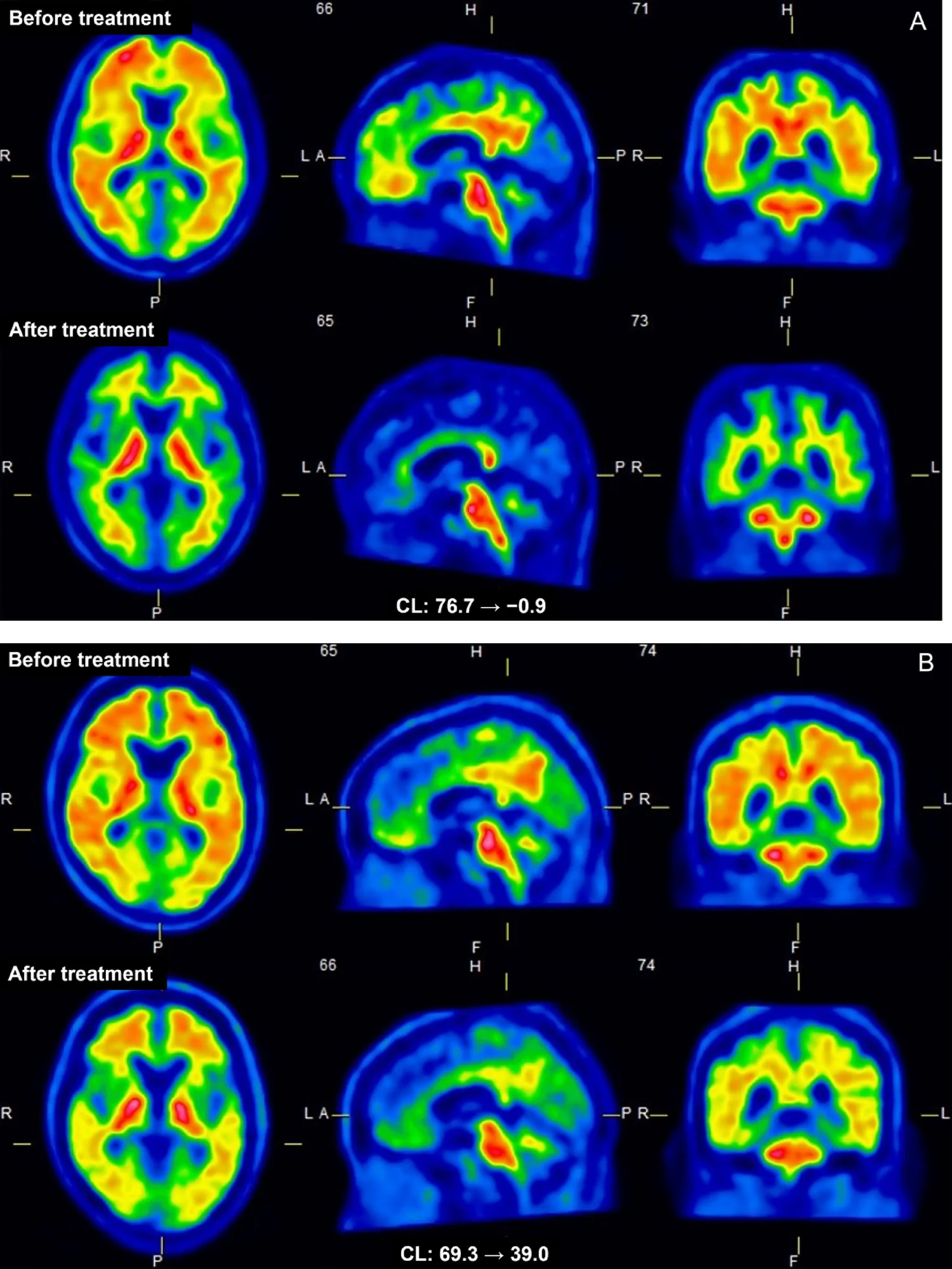
Representative amyloid PET images obtained before and after donanemab treatment. **A** A patient showing conversion from visually amyloid-positive to visually amyloid-negative status, with the Centiloid value decreasing from 76.7 to − 0.9. **B** A patient showing persistent visual amyloid positivity despite substantial reduction in amyloid burden, with the Centiloid value decreasing from 69.3 to 39.0

A total of 20 patients underwent serial amyloid PET imaging before and after donanemab treatment. The mean interval between baseline and follow-up PET scans was 11.7 ± 0.3 months. Centiloid values showed a marked reduction following treatment, decreasing from 52.3 ± 23.4 at baseline to − 5.6 ± 16.5 at follow-up (mean change, − 58.0; *p* < 0.001). All patients demonstrated a reduction in Centiloid values (Fig. 1). Quantitative changes in Centiloid values and tracer-specific SUVr are summarized in Table 1. Tracer-specific analyses of SUVr also revealed consistent decreases after treatment (Fig. 2). In the flutemetamol group (n = 12), pons-referenced SUVr significantly decreased, while in the florbetapir group (n = 8), cerebellar-referenced SUVr also showed significant reductions (both *p* < 0.001). All patients in both tracer groups exhibited a reduction in SUVr. On visual assessment, 17 of 20 patients (85%) converted from amyloid-positive to amyloid-negative status after treatment. Notably, three patients remained amyloid-positive at follow-up despite substantial reductions in Centiloid values, indicating residual amyloid burden in a subset of cases. Representative amyloid PET images are shown in Fig. 3. Figure 3A demonstrates a typical case with conversion to amyloid-negative status, whereas Fig. 3B illustrates persistent visual amyloid positivity despite a substantial reduction in amyloid burden. Regional SUVr analyses derived from CortexID are provided in Supplementary Tables 1 and 2 and offer additional information regarding regional patterns of amyloid reduction.

**Table 1:** Changes in quantitative amyloid PET parameters before and after donanemab treatment

Parameter	Before treatment	After treatment	Mean change	*p* value
Centiloid (n = 20)	52.3 ± 23.4	− 5.6 ± 16.5	− 58.0 ± 20.0	< 0.001
Flutemetamol SUVr (pons reference, n = 12)	0.69 ± 0.09	0.47 ± 0.05	− 0.22 ± 0.09	< 0.001
Florbetapir SUVr (cerebellar reference, n = 8)	1.20 ± 0.10	0.95 ± 0.09	− 0.25 ± 0.06	< 0.001

## Discussion

In this study, we demonstrated a substantial reduction in amyloid burden on serial amyloid PET following donanemab treatment in a real-world clinical setting. Centiloid values significantly decreased in all patients, with a mean reduction of 58.0, and 85% of patients converted from amyloid-positive to amyloid-negative status. These findings indicate a robust therapeutic effect of donanemab that is clearly detectable using quantitative amyloid PET.

Not all patients achieved amyloid-negative status at follow-up, although all demonstrated substantial reductions in amyloid burden. This finding reflects variability in treatment response observed in routine clinical practice and suggests that complete amyloid clearance may not be achieved in all cases within one year. In 2 cases, quantitative measures indicated amyloid levels below the commonly used threshold (approximately 20 Centiloid), whereas visual assessment still suggested residual amyloid positivity. This discrepancy may reflect the fact that quantitative measures capture global amyloid burden, whereas visual assessment can be influenced by focal residual cortical uptake in one or a few regions. In addition, quantitative amyloid PET measurements are known to be affected by factors such as tracer characteristics, reference region selection, and image acquisition and processing parameters [15], which may further contribute to the observed discordance between quantitative and visual assessments. Furthermore, regional heterogeneity in the rate of amyloid reduction may also play a role, as differences in clearance across brain regions could result in persistent focal uptake despite overall reduction in global quantitative measures. Another possible explanation is that visual interpretation may become more challenging after anti-amyloid therapy than in untreated patients. Current visual interpretation criteria were originally developed for diagnostic assessment of untreated individuals and may not fully account for imaging patterns observed after substantial but incomplete amyloid removal. In this setting, readers may adopt a relatively conservative approach and classify scans as positive when focal residual cortical uptake remains visible, even when global quantitative measures indicate a marked reduction in amyloid burden. These findings suggest that visual and quantitative assessments may provide complementary information during treatment monitoring.

The amyloid-lowering effect of donanemab has been well established in clinical trials, including the TRAILBLAZER studies, which reported substantial reductions in Centiloid values. Recent analyses have further emphasized the importance of posttreatment amyloid levels in relation to clinical outcomes [16]. However, clinical data on serial amyloid PET changes remain limited. Our results confirm that these treatment effects are consistently observed in routine clinical practice, supporting the clinical utility of amyloid PET as a biomarker for treatment response. These findings are in agreement with clinical trial results and further support the reproducibility of amyloid PET in routine clinical practice. Unlike clinical trials conducted under highly standardized conditions, our study reflects routine clinical practice, including heterogeneous patient populations, the use of two different amyloid PET tracers, and quantitative assessment using clinically available software. Despite these real-world variations, substantial reductions in amyloid burden were consistently observed across all patients. This supports the robustness and clinical applicability of amyloid PET for monitoring treatment response in everyday practice.

An important strength of this study is the use of Centiloid scaling, which enables direct comparison across different tracers. Because SUVr depend on tracer-specific reference regions, pooled analysis of SUVr across tracers is not appropriate. In this study, SUVr was therefore analyzed separately for each tracer, while Centiloid values were used as the primary metric. Notably, both flutemetamol (pons-referenced) and florbetapir (cerebellar-referenced) demonstrated consistent reductions in SUVr, further supporting the robustness of the observed treatment effect.

Another notable finding is the high rate of conversion to amyloid-negative status. This has important clinical implications, as amyloid PET negativity is increasingly considered a potential therapeutic goal in anti-amyloid treatment strategies. Our results suggest that substantial amyloid clearance can be achieved in routine clinical settings, which may have implications for treatment monitoring and decision-making.

Although the overall trend of amyloid reduction may appear expected based on prior clinical trials, the uniformity and magnitude of change observed in this real-world cohort are noteworthy. In particular, all patients showed a reduction in quantitative measures. This finding further supports the robustness of amyloid PET in detecting treatment-related changes and highlights its potential as a quantitative biomarker for evaluating therapeutic response.

This study has several limitations. First, the sample size was relatively small, and the study was conducted at a single institution. Second, the follow-up period was limited, and long-term changes in amyloid burden were not assessed. Third, clinical outcomes such as cognitive function were not systematically analyzed, and therefore the relationship between amyloid reduction and clinical improvement remains unclear. Fourth, the use of different tracers and analysis software may have introduced additional variability.

## Conclusion

Serial amyloid PET demonstrated a marked and consistent reduction in amyloid burden following donanemab treatment in this real-world cohort. These findings highlight the robustness and reproducibility of quantitative amyloid PET for assessing treatment response in routine clinical practice.

## Supplementary Information

Below is the link to the electronic supplementary material.

Supplementary file1.

Supplementary file2.
