# Supplementary material for: Serial amyloid PET demonstrates marked reduction in amyloid burden following donanemab treatment: a real-world cohort study
Source: Jpn J Radiol. 2026 Jul 27;44(11):2012–8. doi: 10.1007/s11604-026-02054-x (PMC13722638; doi:10.1007/s11604-026-02054-x)
Supplement: Supplementary file 1 [file 11604_2026_2054_MOESM1_ESM.docx]

Supplementary Table 1

Regional SUVr changes derived from CortexID in patients undergoing ^18^F-flutemetamol PET before and after donanemab treatment.

| Region | Baseline SUVr | Follow-up SUVr | Mean Change | p value |
| --- | --- | --- | --- | --- |
| Composite | 0.69 ± 0.09 | 0.47 ± 0.05 | -0.22 ± 0.09 | <0.001 |
| Prefrontal R | 0.68 ± 0.11 | 0.43 ± 0.06 | -0.25 ± 0.11 | <0.001 |
| Prefrontal L | 0.67 ± 0.10 | 0.43 ± 0.05 | -0.24 ± 0.10 | <0.001 |
| Anterior Cingulate R | 0.67 ± 0.11 | 0.44 ± 0.07 | -0.23 ± 0.12 | <0.001 |
| Anterior Cingulate L | 0.68 ± 0.11 | 0.45 ± 0.06 | -0.23 ± 0.12 | <0.001 |
| Precuneus PostCing R | 0.73 ± 0.09 | 0.48 ± 0.08 | -0.25 ± 0.10 | <0.001 |
| Precuneus PostCing L | 0.76 ± 0.12 | 0.51 ± 0.08 | -0.25 ± 0.11 | <0.001 |
| Parietal R | 0.71 ± 0.10 | 0.50 ± 0.06 | -0.22 ± 0.09 | <0.001 |
| Parietal L | 0.68 ± 0.10 | 0.48 ± 0.07 | -0.20 ± 0.10 | <0.001 |
| Temporal Lateral R | 0.73 ± 0.08 | 0.53 ± 0.05 | -0.19 ± 0.08 | <0.001 |
| Temporal Lateral L | 0.68 ± 0.08 | 0.51 ± 0.05 | -0.17 ± 0.08 | <0.001 |
| Occipital R | 0.65 ± 0.10 | 0.54 ± 0.05 | -0.11 ± 0.07 | <0.001 |
| Occipital L | 0.66 ± 0.11 | 0.55 ± 0.05 | -0.11 ± 0.09 | 0.001 |
| Sensorimotor R | 0.61 ± 0.08 | 0.47 ± 0.05 | -0.14 ± 0.06 | <0.001 |
| Sensorimotor L | 0.60 ± 0.09 | 0.47 ± 0.06 | -0.13 ± 0.07 | <0.001 |
| Temporal Mesial R | 0.49 ± 0.05 | 0.41 ± 0.03 | -0.08 ± 0.03 | <0.001 |
| Temporal Mesial L | 0.48 ± 0.05 | 0.42 ± 0.02 | -0.07 ± 0.04 | <0.001 |
