# Supplementary material for: Serial amyloid PET demonstrates marked reduction in amyloid burden following donanemab treatment: a real-world cohort study
Source: Jpn J Radiol. 2026 Jul 27;44(11):2012–8. doi: 10.1007/s11604-026-02054-x (PMC13722638; doi:10.1007/s11604-026-02054-x)
Supplement: Supplementary file 2 [file 11604_2026_2054_MOESM2_ESM.docx]

Supplementary Table 2

Regional SUVr changes derived from CortexID in patients undergoing ^18^F-florbetapir PET before and after donanemab treatment.

| Region | Baseline SUVr | Follow-up SUVr | Mean Change | p value |
| --- | --- | --- | --- | --- |
| Composite | 1.20 ± 0.10 | 0.95 ± 0.09 | -0.25 ± 0.06 | <0.001 |
| Prefrontal R | 1.11 ± 0.16 | 0.86 ± 0.10 | -0.25 ± 0.10 | <0.001 |
| Prefrontal L | 1.12 ± 0.11 | 0.85 ± 0.09 | -0.27 ± 0.06 | <0.001 |
| Anterior Cingulate R | 1.16 ± 0.15 | 0.89 ± 0.09 | -0.27 ± 0.12 | <0.001 |
| Anterior Cingulate L | 1.21 ± 0.15 | 0.95 ± 0.11 | -0.27 ± 0.08 | <0.001 |
| Precuneus PostCing R | 1.26 ± 0.08 | 0.95 ± 0.07 | -0.31 ± 0.07 | <0.001 |
| Precuneus PostCing L | 1.37 ± 0.05 | 1.01 ± 0.06 | -0.35 ± 0.04 | <0.001 |
| Parietal R | 1.25 ± 0.12 | 0.99 ± 0.11 | -0.25 ± 0.06 | <0.001 |
| Parietal L | 1.23 ± 0.10 | 0.96 ± 0.09 | -0.28 ± 0.07 | <0.001 |
| Temporal Lateral R | 1.26 ± 0.13 | 1.08 ± 0.12 | -0.18 ± 0.07 | <0.001 |
| Temporal Lateral L | 1.25 ± 0.09 | 1.05 ± 0.10 | -0.20 ± 0.06 | <0.001 |
| Occipital R | 1.23 ± 0.15 | 1.15 ± 0.14 | -0.09 ± 0.08 | 0.017 |
| Occipital L | 1.31 ± 0.15 | 1.17 ± 0.13 | -0.13 ± 0.07 | <0.001 |
| Sensorimotor R | 1.07 ± 0.16 | 0.93 ± 0.11 | -0.14 ± 0.09 | 0.003 |
| Sensorimotor L | 1.06 ± 0.15 | 0.92 ± 0.11 | -0.13 ± 0.06 | <0.001 |
| Temporal Mesial R | 0.96 ± 0.08 | 0.90 ± 0.08 | -0.06 ± 0.02 | <0.001 |
| Temporal Mesial L | 0.98 ± 0.09 | 0.92 ± 0.09 | -0.07 ± 0.02 | <0.001 |
